# Randomized Controlled Trial of Fish Oil and Montelukast and Their Combination on Airway Inflammation and Hyperpnea-Induced Bronchoconstriction

**DOI:** 10.1371/journal.pone.0013487

**Published:** 2010-10-18

**Authors:** Sandra Tecklenburg-Lund, Timothy D. Mickleborough, Louise A. Turner, Alyce D. Fly, Joel M. Stager, Gregory S. Montgomery

**Affiliations:** 1 Human Performance and Exercise Biochemistry Laboratory, Department of Kinesiology, Indiana University, Bloomington, Indiana, United States of America; 2 Department of Applied Health Science, Indiana University, Bloomington, Indiana, United States of America; 3 Indiana University School of Medicine, Indiana University, Indianapolis, Indiana, United States of America; 4 Health and Human Performance, Nebraska Wesleyan University, Lincoln, Nebraska, United States of America; University of Giessen Lung Center, Germany

## Abstract

**Background:**

Both fish oil and montelukast have been shown to reduce the severity of exercise-induced bronchoconstriction (EIB). The purpose of this study was to compare the effects of fish oil and montelukast, alone and in combination, on airway inflammation and bronchoconstriction induced by eucapnic voluntary hyperpnea (EVH) in asthmatics.

**Methods:**

In this model of EIB, twenty asthmatic subjects with documented hyperpnea-induced bronchoconstriction (HIB) entered a randomized double-blind trial. All subjects entered on their usual diet (pre-treatment, n = 20) and then were randomly assigned to receive either one active 10 mg montelukast tablet and 10 placebo fish oil capsules (n = 10) or one placebo montelukast tablet and 10 active fish oil capsules totaling 3.2 g EPA and 2.0 g DHA (n = 10) taken daily for 3-wk. Thereafter, all subjects (combination treatment; n = 20) underwent another 3-wk treatment period consisting of a 10 mg active montelukast tablet or 10 active fish oil capsules taken daily.

**Results:**

While HIB was significantly inhibited (p<0.05) by montelukast, fish oil and combination treatment compared to pre-treatment, there was no significant difference (p>0.017) between treatment groups; percent fall in forced expiratory volume in 1-sec was −18.4±2.1%, −9.3±2.8%, −11.6±2.8% and −10.8±1.7% on usual diet (pre-treatment), fish oil, montelukast and combination treatment respectively. All three treatments were associated with a significant reduction (p<0.05) in F_E_NO, exhaled breathe condensate pH and cysteinyl-leukotrienes, while the fish oil and combination treatment significantly reduced (p<0.05) urinary 9α, 11β-prostaglandin F_2_ after EVH compared to the usual diet; however, there was no significant difference (p>0.017) in these biomarkers between treatments.

**Conclusion:**

While fish oil and montelukast are both effective in attenuating airway inflammation and HIB, combining fish oil with montelukast did not confer a greater protective effect than either intervention alone. Fish oil supplementation should be considered as an alternative treatment for EIB.

**Trial Registration:**

ClinicalTrials.gov NCT00676468

## Introduction

Exercise-induced bronchoconstriction (EIB) and exercise-induced asthma are terms used interchangeably that describe a transient narrowing of the airways during or following exercise [1] that can occur in patients with asthma [2] and elite athletes [3]. The mechanisms responsible for EIB likely involve multiple mechanistic pathways, however it is generally accepted that exercise or dry air hyperpnea play an important role as an initiating stimulus through airway surface effects of water loss, which include mucosal cooling and dehydration [1]. This transient dehydration causes an increase in airway surface liquid osmolarity which activates histamine, neuropeptides, and arachidonic acid metabolites such as cysteinyl (Cyst)- leukotrienes [(LTs) e.g., LTC_4_, D_4_ and E_4_)] and prostaglandins, from resident airway cells, resulting in bronchial smooth muscle contraction and subsequent airway obstruction [4].

The Cyst-LTs, produced predominantly by mast cells and eosinophils [5], are the most potent constrictors of human airway smooth muscle *in vitro* and *in vivo* [5], [6], and have been implicated in the pathophysiology of airway hyperresponsiveness and, subsequently in airway remodeling [7]. There are distinct approaches to reducing the actions of Cyst-LTs: inhibition of biosynthesis (e.g., inhibitors of 5-lipoxygenase (5-LO) and 5-LO-activating protein) or blockade of Cyst-LT_1_ receptors using leukotriene receptor antagonists [e.g., montelukast sodium (Singulair ®) and zafirlukast] [8]. Both these drugs inhibit the maximum bronchonstrictor response to exercise or dry air hyperpnea by 30 to 70% [9], [10], [11], [12], [13], [14], which indicates that other mediators may play a role in the pathogenesis of EIB [10]. Whilst both drugs have been shown to reduce EIB severity, only montelukast has been approved by the US Food and Drug Administration for this purpose [15].

Although the treatment of EIB almost exclusively involves pharmacotherapy, there is now convincing evidence that dietary modification has the potential to reduce the severity of this condition [16]. While the clinical data on the effect of fish oil supplementation in asthma has been equivocal [17] supplementing the diet with fish oil, rich in omega-3 (n-3) polyunsaturated fatty acids (PUFA), such as eicosapentaenoic acid (EPA) and docosahexaenoic acid (DHA), in individuals with EIB has yielded promising results [18], [19]. Eicosapentaenoic acid can compete with arachidonic acid, as a substrate for cyclooxygenase (COX)-2 and 5-lipoxygenase (5-LO) enzymes and be converted to less inflammatory leukotrienes and prostanoids [20], [21]. At present the mechanism(s) underpinning the anti-inflammatory effects of DHA are unclear, but may be related to altered gene transcription and translation via direct or indirect actions on intracellular signaling pathways [22], [23].

Since the Cyst-LTs are, overall, the most potent pro-inflammatory mediators causing EIB [24] an important question is how dietary fish oil supplementation fits in with the available armamentarium (e.g., Cyst-LT type 1 receptor antagonists) to decrease the expression of Cyst-LTs, and whether fish oil supplementation may be additive, or used in its own right to block the bronchoconstrictor response. Therefore, the primary aim of the present study was to evaluate the effects of fish oil supplementation and a Cyst- LT_1_ receptor antagonist [montelukast sodium (Singulair®), a commonly used medication to treat EIB], alone and in combination, on airway inflammation and the bronchoconstrictor response to dry air hyperpnea in individuals with asthma.

It was hypothesized that both fish oil and montelukast would be effective in attenuating hyperpnea-induced bronchoconstriction (HIB) and airway inflammation, and that the two treatments combined would infer even greater protection against HIB than either intervention alone. Our findings showed that fish oil supplementation and montelukast are both effective in moderating airway inflammation and HIB in asthmatic individuals. However, combining fish oil with montelukast did not confer a greater protective effect than either intervention alone.

## Methods

The protocol for this trial and supporting CONSORT checklist are available as supporting information; see Checklist S1 and Protocol S1.

### Subjects

The study was conducted between October 2008 and March 2009 in the United States. Twenty subjects aged 18 to 27 years old with both physician-diagnosed asthma and documented hyperpnea-induced bronchoconstriction were recruited from a population of university students and the local community. Subject characteristics at baseline are presented in Table 1. All subjects had clinically treated mild to moderate persistent asthma, with an resting forced expiratory volume in 1-sec (FEV_1_) of >65% predicted (Table 2), and EIB as demonstrated by a greater than 10% drop in FEV_1_ following a eucapnic voluntary hyperventilation (EVH) challenge [25]. A group of non-asthmatic (control) subjects was not included in the present study, as it has been shown that fish oil supplementation does not alter pulmonary function or inflammatory mediator generation in this population [19].

**Table 1:** Subject (baseline) characteristics.

Characteristics (n = 20)	
Sex, n	
Males	6
Females	14
Age, yr (range)	20.8±2.2 (18–27)
Height, m	1.70±0.04
Weight, kg	72.4±13.4
BMI, kg/m2	24.9±4.0
Morning peak flow, L/min	380.7±82.7
Evening peak flow, L/min	393.1±90.7

Values reported are mean ± SEM.

**Table 2:** Pre-hyperpnea (baseline) pulmonary function.

Treatment Period				
	Pre-treatment (n = 20)	Montelukast (n = 10)	Fish Oil (n = 10)	Combination Treatment (n = 20)
FVC (L)	4.51±0.22	4.30±0.18	4.64±0.43	4.61±0.31
% predicted	105.0±3.3%	100.9±4.4%	106.6±5.7%	105.7±3.9%
FEV_1_ (L)	3.67±0.19	3.34±0.19	3.81±0.31	3.69±0.24
% predicted	100.8±4.2%	96.1±6.3%	102.5±5.6%	102.3±5.0%
FEF_25–75%_ (L/min)	3.67±0.27	3.00±0.26	3.82±0.38	3.71±0.29
% predicted	90.6±6.3%	80.7±6.4%	100.3±8.8%	91.7±6.8%

Values reported are mean ± SEM.

All subjects had a history of shortness of breath, chest tightness, and intermittent wheezing following exercise, which was relieved by bronchodilator therapy. No subjects who volunteered for the study were currently taking any maintenance medications (e.g., corticosteroids and leukotriene modifiers) for asthma. Short acting β2-agonists were discontinued 12 h prior to testing. Caffeine/alcohol and physical exercise was not permitted 8 h and 12 h respectively prior to the EVH challenge. Subjects were also excluded if they had a history of taking fish oil supplements and regularly consumed more than one fish meal per week. Subjects were asked not to eat more than one fish meal per week during the course of the study. Subjects were excluded if they were pregnant, had a history of hyperlipidemia, hypertension, diabetes, bleeding disorders, or delayed clotting time. The study was approved by the Indiana University Institutional Review Board for Human Subjects, and written informed consent for all subjects was obtained prior to participation in the study. The study was registered as a phase 1 clinical trial with clinical trials.gov (study # NCT00676468).

### Study Design

The study was conducted over the course of 8 weeks as a matched (by gender), double-blind randomized controlled parallel group trial over 3 weeks followed by a single group trial (both groups combined) for a further 3 weeks. Each subject underwent a 2 –week run period preceding the start of the trial in which asthma symptoms were recorded. Testing in the laboratory was conducted following the run in period (usual diet) and at the conclusion of each 3-week treatment period. All tests were conducted in the human performance and exercise biochemistry laboratories at Indiana University-Bloomington. An independent investigator having no contact with the subjects and no involvement in data collection or analysis used a computerized random number generator (“research randomizer” http://www.randomizer.org/form.htm) to create the randomization sequence which was stratified by sex with a 1∶1 allocation using a fixed random block size of two.

All subjects entered the study on their usual diet (*phase 1*) and then randomly assigned to one of two groups matched by gender (Figure S1). The two parallel treatment regimens (*phase 2*) consisted of either one active montelukast sodium (Singulair®; Merck& Co. Inc, Whitehouse Station, NJ) 10 mg tablet taken orally once daily and 10 placebo fish oil capsules (Nordic Naturals, Watsonville, CA) per day containing soybean oil (n = 10) or one placebo montelukast sodium tablet taken orally once daily and 10 active fish oil capsules taken daily equaling a total of 3.2 g EPA and 2.0 g DHA (n = 10). Following *phase 2*, all subjects underwent another 3-week treatment period consisting of an active montelukast sodium 10 mg tablet, taken orally once daily in the evening and 10 active fish oil capsules taken daily (n = 20) (*phase 3*). Both the active and placebo fish oil capsules, and the active and placebo montelukast tablets, were identical in size and appearance to each other.

A eucapnic voluntary hyperventilation (EVH) challenge (surrogate for an exercise challenge test) was performed at the end of phase 1, 2 and 3 [26]. Pulmonary function was monitored pre-EVH and post-EVH at 5, 10, 15, and 20 min. Exhaled nitric oxide (F_E_NO), a non-invasive measure of airway inflammation, was measured pre-EVH and at 30 min post-EVH. Exhaled breath condensate (EBC) was collected pre-EVH and post-EVH from 0–10 min and analyzed for the presence of the Cyst-LTs and airway pH. Urine samples were collected pre-EVH and 60 min post-EVH for 9α, 11β-Prostaglanind F_2_ analysis. Subjects were asked to record daily peak flow and symptom scores throughout the course of the study. Food frequency questionnaires were administered at the beginning of each testing session.

### Eucapnic Voluntary Hyperventilation

The EVH protocol required subjects to breathe compressed dry air (<3 mg H_2_O.L^−1^ air and 21%O_2_, 5%CO_2_, balance N_2_) at a predetermined rate of 85% of maximal voluntary ventilation (estimated from 30× the volume of resting FEV_1_) for 6 min [13]. Gas flowed from a cylinder to a reservoir bag through high-pressure tubing. From the reservoir bag gas was directed to the subject through a tube connected to a two way breathing valve and mouthpiece [13]. Expired gases passed through a flow sensor and ventilation was measured and recorded as verification of respiration intensity (Vmax 22 Metabolic Measurement Cart, SensorMedics, Yorba Linda, CA). EVH has a relatively high sensitivity to identify EIB and a high negative-predictive value [27].

### Pulmonary Function Tests

Pulmonary function tests were conducted on all subjects using a calibrated computerized pneumotachograph spirometer (Vmax 22, SensorMedics, Yorba Linda, CA) according to according to American Thoracic Society (ATS) recommendations [28]. The maximum percentage fall in FEV_1_ from the baseline (pre-EVH) value was calculated using the following equation: (Pre-EVH FEV_1_ – lowest post-EVH FEV_1_)/(Pre-EVH FEV_1_). In addition, the bronchoconstrictor response to EVH was assessed as the area under the curve of the percentage fall in post-EVH FEV_1_ plotted against time for 20 min (AUC_0–20_), using trapezoidal integration.

### Fraction of Exhaled Nitric Oxide

Fraction of exhaled nitric oxide (F_E_NO) was measured with an online measurement of resting values using a restricted exhaled breath protocol (NOA 280i Nitric Oxide Analyzer, Accurate NO Breath Kit, Thermal Mass Flowmeter, NO Analysis software Version 3.21, Sievers Instruments, Boulder, CO). Measurements were conducted as outlined by American Thoracic Society guidelines [29]. Three exhalations were performed with nose clips at each test with at least 30s between exhalations [29]. The procedure entailed maximal inhalation to total lung capacity and immediate exhalation against expiratory resistance for at least 6 sec to obtain a NO plateau lasting at least 3 sec. Subjects were instructed to maintain a flow rate of 50±10 mL/s as monitored by a visual computer display.

### Quantification of Exhaled Breath Condensate Markers

EBC samples were collected with a specially designed condensing chamber (ECoScreen, Jaeger, Hoechberg, Germany) using ATS/ERS recommendations [30]. The EBC protocol required subjects to breathe normally, wearing nose clips, through a mouthpiece connected to a non-rebreathing valve, whereby exhaled breath entered a condenser system. A temperature of −20°C inside the condensing chamber, which was maintained throughout the collection time, produced immediate sample freezing. Exhaled breath was collected for 10 min prior to and intervals (0–10min) following the EVH challenge.

The pH of the non-deaerated EBC was measured immediately following collection (Orion 2 star pH meter, Thermo Scientific, Beverly, MA). It has been shown that pH measurements in EBC collected by ECoScreen are repeatable and reproducible [31].

The remainder of the condensate was stored at −80°C for later analysis of Cyst-LTs using enzyme immunoassay techniques (Cayman Chemicals, Ann Arbor, MI) as previously discussed [18]. Cross-reactivity of the Cyst-LT antibody against an array of related compounds was: LTC_4_ (100%), LTD_4_ (100%), LTE_4_ (67%), LTD_5_ (61%), LTC_5_ (54%), LTE_5_ (41%), N-acetyl-LTE_4_ (10.5%) and below 0.01% for other primary eicosanoid metabolites. The intra- and inter-assay coefficient of variation (CV) for the Cyst-LT enzyme immunoassay kit was <10% respectively.

### Urinary 9α, 11β- Prostaglandin (PG) F_2_ Quantification

Urine collection containers were provided to each subject during each visit to the laboratory, and the urine was subsequently pipetted into microfuge tubes and stored at −80°C until analysis. Urine was assayed for 9α,11β-PGF_2_ using enzyme immunoassay techniques (Cayman Chemicals, Ann Arbor, MI) as discussed previously [19]. The 9α,11β-PGF_2_ antibody cross-reacted with 2,3 dinor-11β-PGF_2α_ (10%), 11β-13,14-dihydro-15-keto-PGF_2α_ (0.5%) and below 0.01% for all other primary eicosanoid metabolites. Inter- and intra-assay CV for the 9α,11β-PGF_2_ assay were <15%. The concentration of 9α,11β-PGF_2_ was adjusted for creatinine concentration (Cayman Chemicals, Ann Arbor, MI). The intra- and inter assay CV for creatinine was 2.7 and 3.0 respectively.

### Nutrient Intake and Compliance

Nutrient intake was monitored to ensure that dietary factors that could potentially affect asthma or EIB did not change through the course of the study. Nutrient data was collected using the GSEL food frequency questionnaire developed by the Nutrition Assessment Shared Resource (NASR) of Fred Hutchinson Cancer Research Center. This questionnaire has been shown to be valid and reliable in the collection of dietary data [32]. Subjects completed the GSEL version of the questionnaire at the first testing session and at the end of each supplementation period. Analysis of GSEL for nutrient intake was conducted by the Fred Hutchinson Cancer Research Center. Nutrients of interest obtained from the GSEL analysis included macronutrient composition, antioxidants,(α-tocopherol, β-carotene, lycopene, Vitamin C), certain minerals (magnesium, sodium, zinc), and types of dietary fatty acids (omega-3, total polyunsaturated fatty acids, saturated fatty acids). Adherence to the treatment regimen was monitored by asking the subjects to document the dose of capsules consumed daily and to return any unused capsules [33]. For the purpose of the study a compliance of ≥90% was considered acceptable.

### Symptoms, Rescue β-Agonist Use and Peak Flow Measurements

Symptoms such as wheeze, shortness of breath, chest tightness, and cough were recorded by each subject once daily with the use of a logbook. The symptom severity rating scale was defined as follows: 0: absent, no symptoms; 1: mild, symptom was minimally troublesome (not sufficient to interfere with normal daily activity or sleep); 2: moderate, symptom was sufficiently troublesome to interfere with normal daily activity or sleep; 3: severe, symptom was so severe as to prevent normal activity and/or sleep. All subjects were instructed to record each use of their rescue medication throughout the study duration, entering each medication use and the number of puffs used per occasion. Subjects were asked to perform 3 peak flow maneuvers at home in the morning and evening 2 weeks prior to the start of study, and throughout the course of the study. The subjects were provided with a peak flow meter (Piko-1, Ferraris, Louisville, CO) and a log to record the best of 3 trials.

### Data Analysis

The primary analysis was on an intention-to-treat basis and involved all subjects who were randomly assigned. Data were analyzed using the SPSS version 16.0 statistical software (SPSS Inc., Chicago, USA). Normality of data was assessed using a Kolmogorov–Smirnov test and Levene's test was used to check for homogeneity of variance between groups. A repeated measures analysis of variance (ANOVA) was used to analyze the data (within-subject) at baseline (n = 10) and for the single (n = 10) and combination treatment (n = 10). A Mauchly's test was conducted to determine if sphericity was violated. If sphericity was violated, the repeated measures ANOVA was corrected using a Greenhouse-Geisser adjustment factor. Pairwise comparisons, with a Bonferroni adjustment (used to maintain an overall type-I error rate of 5%), were used to isolate differences in group means: dividing the probability value (0.05) by the number of pairwise comparisons to be made. As a check, differences between baseline (n = 20) and the combination treatment (n = 20) values were compared using a paired t-test, while an unpaired t-test was used to analyze differences between the two single treatment arms. The Wilcoxon signed rank test was used to compare symptom scores and bronchodilator use during the trial. Data are expressed as mean ± SD.

## Results

### Subjects

Bronchodilator use (average number of doses/puffs per day) was significantly reduced (p<0.05) during the three treatment periods (fish oil: 0.5±0.8; montelukast: 0.4±0.5; combination therapy (fish oil plus montelukast): 0.3±0.6) compared to pre-treatment (baseline) (0.6±0.9). No significant difference (p>0.017) in bronchodilator use was observed between the three treatment periods. No significant difference (p>0.05) was found between pre-treatment (0.7±0.1) and following treatment with fish oil (0.7±0.2), montelukast (0.6±0.2) and combination therapy (0.6±0.1) for asthma symptoms scores. In addition, no significant difference (p>0.017) was found for asthma symptom scores between three treatments. No significant differences (p<0.017) were found in morning or evening peak flow (morning: 403.2±24.1 L/min, pre-supplementation; 379.8±21.4 L/min, montelukast; 357.8±33.9 L/min, fish oil; and 382.2±25.1 L/min combination therapy) (evening: 418±23.3 L/min, pre-supplementation; 392.7±17.3 L/min, montelukast; 368.3±34.9 L/min, fish oil; and 393.3±27.6 L/min combination therapy).

### Pulmonary Function

**Figure 1:**
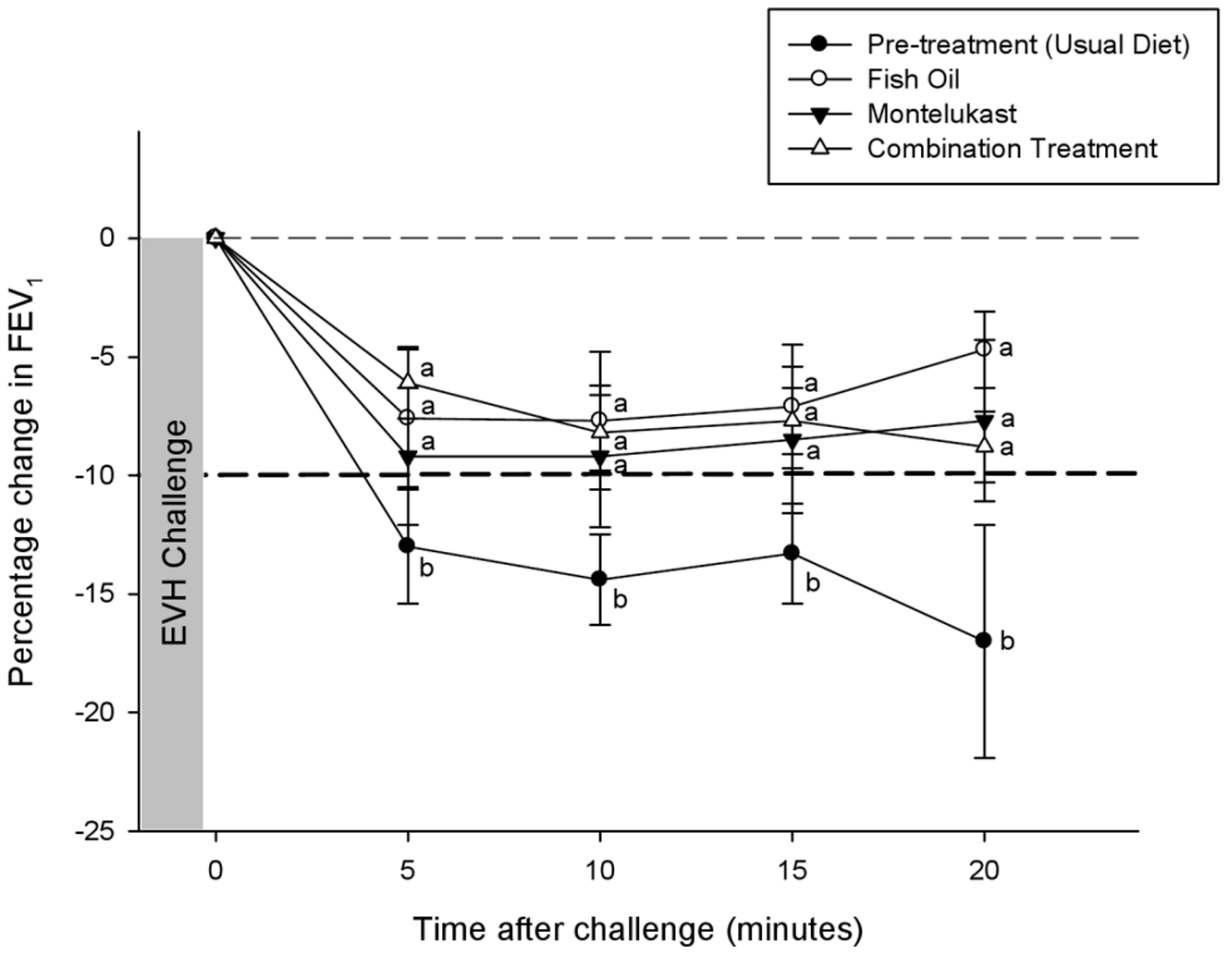
The percentage change in FEV_1_ from pre- to post- EVH for the usual diet (pre-treatment) and across the three treatments. Reductions in post-EVH in excess of 10% represent abnormal pulmonary function. Letters a and b refer to comparisons by treatment within respective time period. Different letters designate a significant difference (p<0.05).

**Figure 2:**
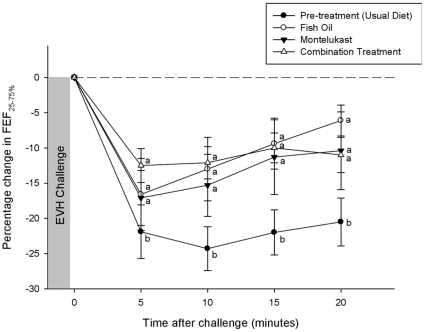
The percentage change in FEF_25–75%_ from pre- to post- EVH for the usual diet (pre-treatment) and across the three treatments. Letters a and b refer to comparisons by treatment within respective time period. Different letters designate a significant difference (p<0.05).

No significant difference (p>0.017) was observed between groups (pre-supplementation, fish oil, montelukast and combination treatment) in baseline (pre-EVH) pulmonary function (Table 2). The percentage change in the pre- and post-treatment drop in post-EVH FEV_1_ is shown in Figure 1. Prior to treatment the maximum percent drop in post-EVH FEV_1_ was −18.4±2.1%. However, there was a significant reduction (p<0.05) in post-EVH FEV_1_ to −9.3±2.8% on fish oil, to −11.6+2.8%, on montelukast, and to −10.8±1.7% on the combination treatment. There was no significant difference (p>0.017) in the maximum percent drop in post-EVH FEV_1_ between the three treatment groups. Similar changes, as a result of treatment, were observed for the maximum percent drop in post-EVH FEF_25–75%_ (Figure 2) and FVC.

The bronchoconstrictor response to EVH, as determined by the AUC_0–20_ for FEV_1_ was significantly greater (p<0.05) during pre-treatment (−228.8±33.3) compared post-treatment (fish oil, −114.9±35.5; montelukast, −152.8±54.4; combination treatment, −125.5±20.6). No significant difference (p>0.017) between the treatment groups was observed for post-EVH AUC_0–20_ for FEV_1_. A similar pattern was observed for the AUC_0–20_ for post-EVH FEF_25–75%_ and FVC.

### Fraction of Exhaled Nitric Oxide

**Figure 3:**
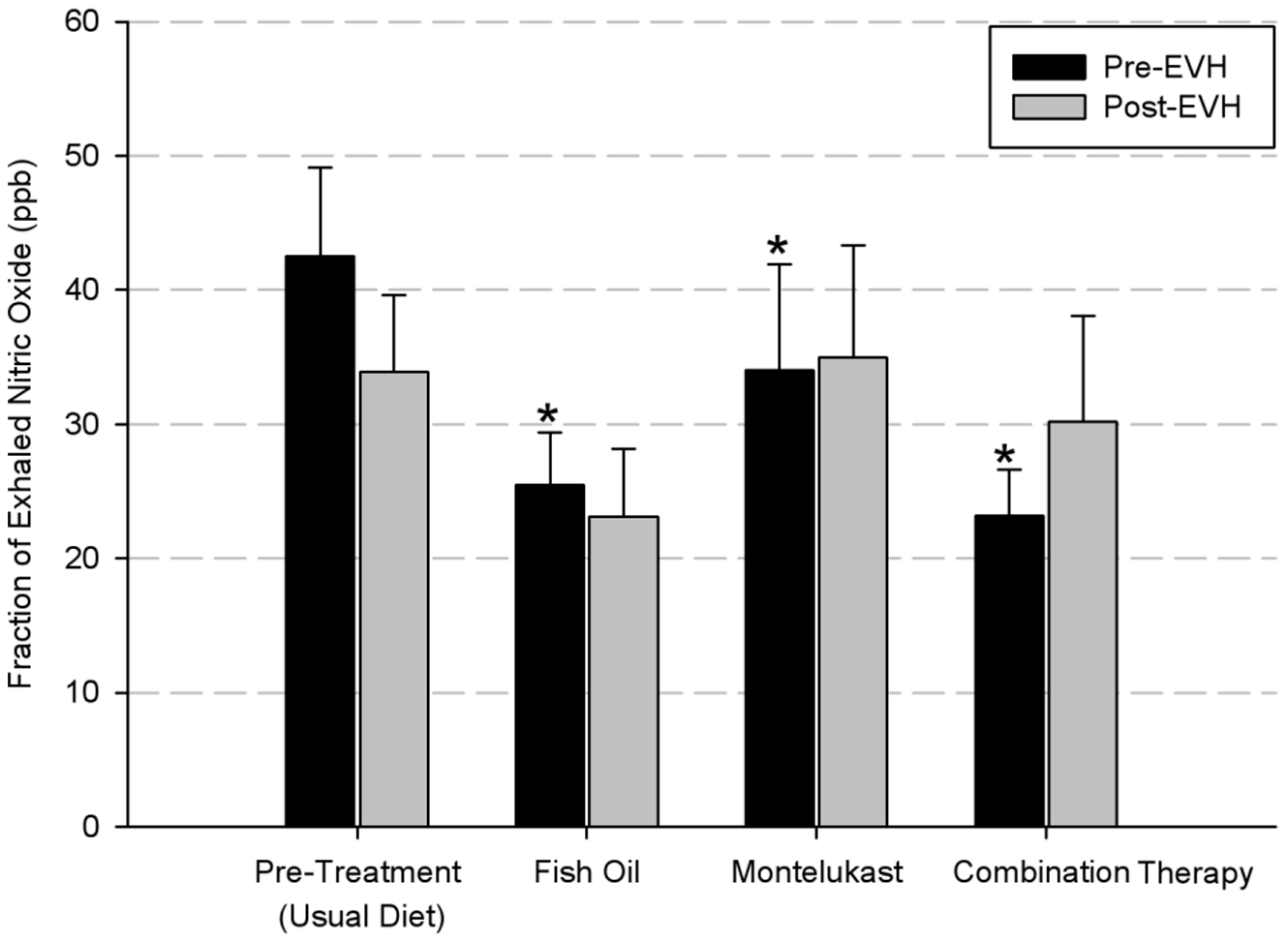
Mean fraction of exhaled nitric oxide (F_E_NO) concentration (ppb). Different letters designate a significant difference (p<0.05) between treatments pre-EVH.

A significant difference (p<0.05) was observed in baseline (pre-EVH) F_E_NO between pre- treatment (42.5±6.6 ppb) and post-treatment values (fish oil, 25.5±3.9 ppb; montelukast, 34.0±7.9 ppb; combination treatment, 23.2±3.4 ppb; Figure 3). However, no significant difference (p>0.017) was observed in baseline F_E_NO between the three treatments. In addition, there were no significant differences (p>0.05) between the pre-treatment (33.9±5.7 ppb) post-EVH F_E_NO compared to post-treatment values (fish oil, 23.1±5.0; montelukast, 35.0±8.3 ppb; combination treatment, 30.2±7.9).

### Exhaled Breath Condensate and Urinary Inflammatory Markers

The EBC pH was significantly lower (p<0.05) pre-supplementation (6.9±0.10) compared to all treatments (fish oil, 7.4±0.11; montelukast, 7.3±0.07; combination treatment, 7.4±0.04). However, the pH of EBC did not differ significantly (p>0.017) between any of the three treatments.

**Figure 4:**
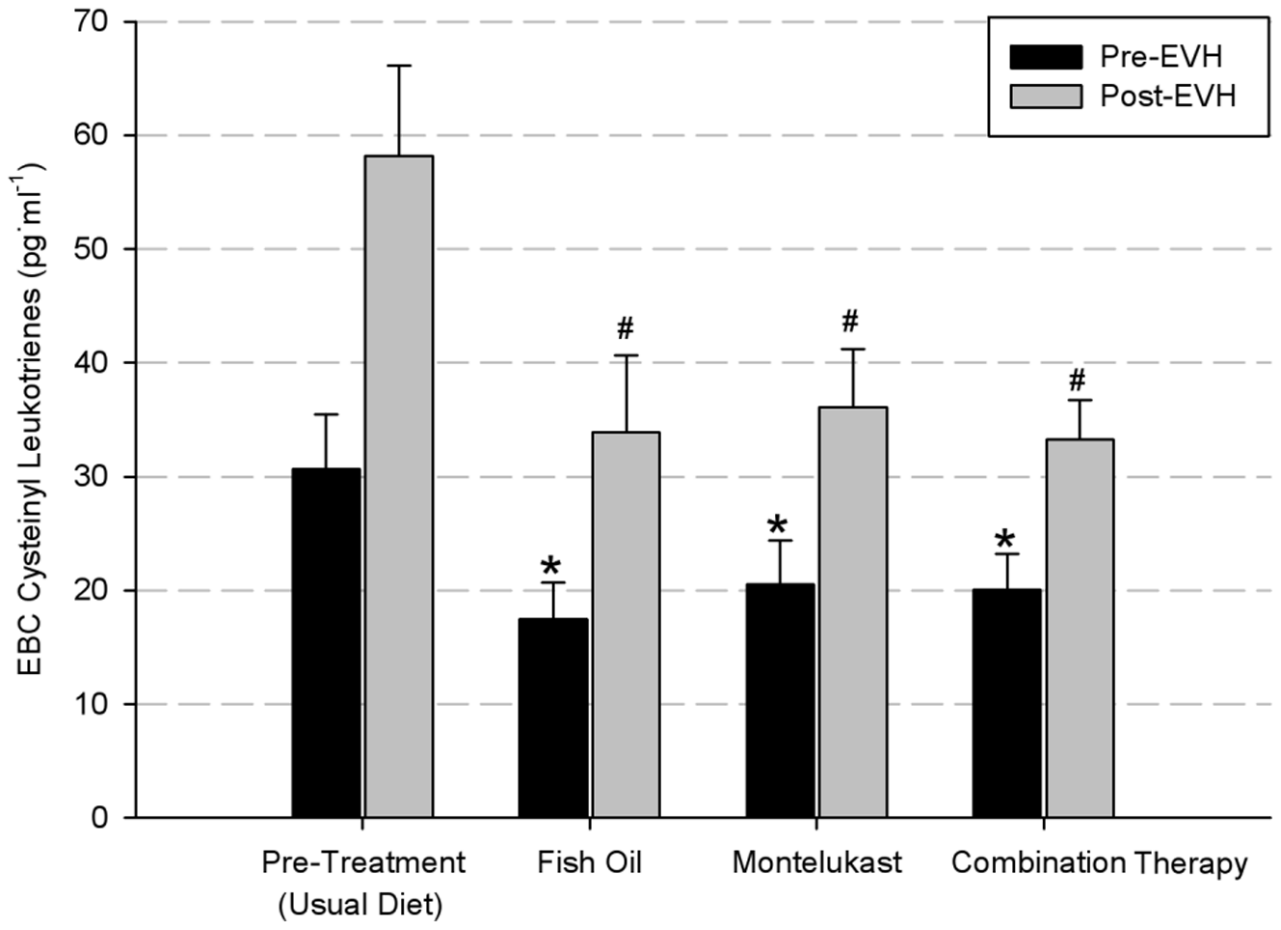
Mean exhaled breathe condensate (EBC) cysteinyl-leukotriene concentration (pg.mg^−1^). * designates a significant difference compared to pre-treatment (usual diet) pre-EVH. # designates a significant difference compared to pre-treatment (usual diet) post-EVH.

Mean EBC Cyst-LT levels for pre- and post-EVH before and after treatment are shown in Figure 4. Fish oil, montelukast and the combination treatment resulted in a significant reduction (p<0.05) in pre-EVH EBC Cyst-LT concentration of 43.0±2.8%, 33.2±2.6% and 34.5±2.3% respectively compared to the pre-EVH Cyst-LT baseline concentration. Likewise, the fish oil, montelukast and combination treatment significantly reduced (p<0.05) the post-EVH EBC Cyst-LT concentration by 41.8±3.4%, 38.0±3.9% and 42.8±2.8% compared to the pre-treatment post-EVH Cyst-LT concentration.

**Figure 5:**
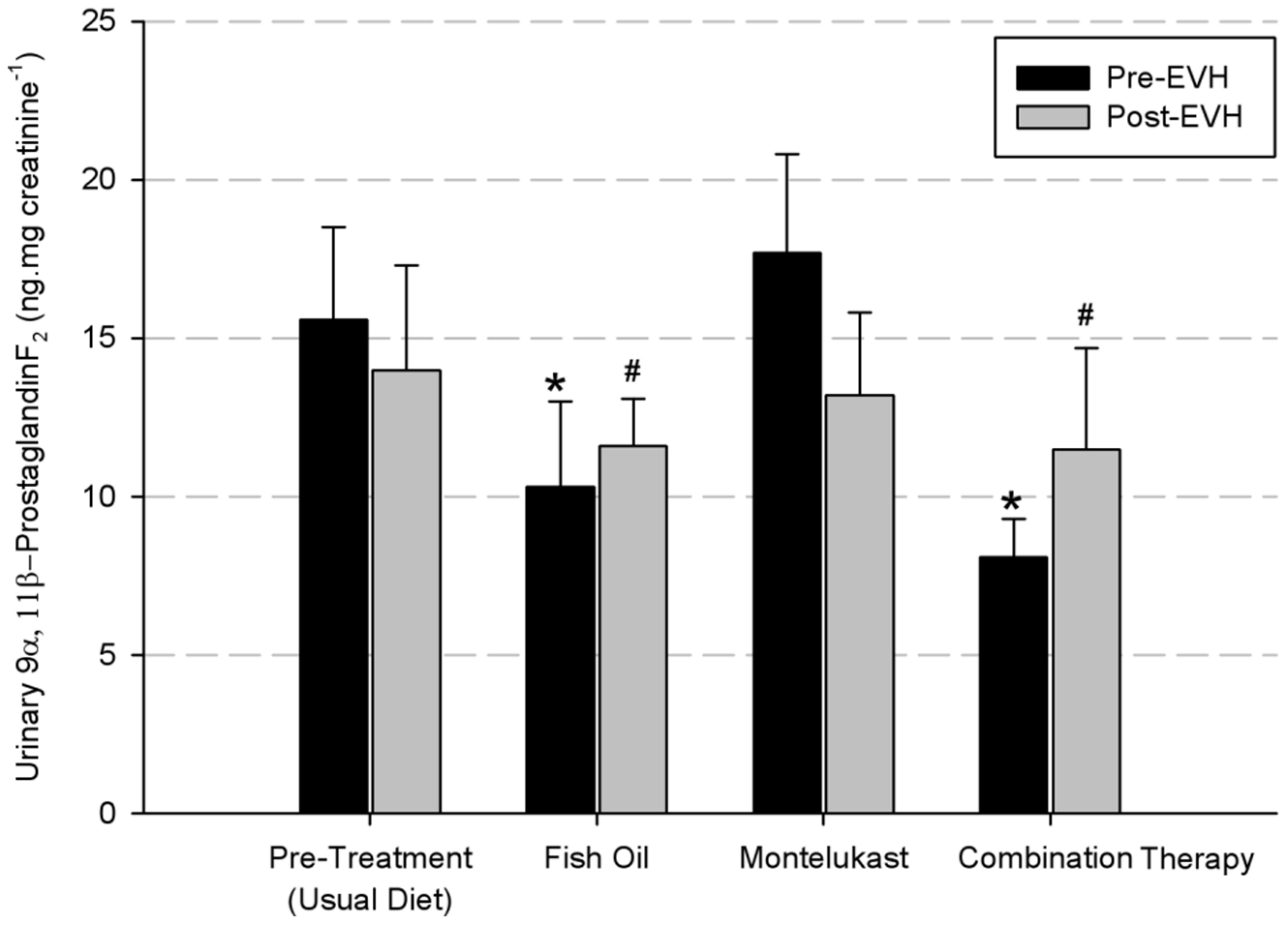
Mean urinary 9α, 11β-prostaglandin F_2_ concentration (ng.mg mmol creatinine^−1^). * designates a significant difference compared to pre-treatment (usual diet) pre-EVH. # designates a significant difference compared to pre-treatment (usual diet) post-EVH.

Mean urinary 9α, 11β PGF_2_ levels for pre- and post-EVH before and after treatment are shown in Figure 5. The fish oil and the combination treatment resulted in a significant reduction (p<0.05) in pre-EVH urinary 9α, 11β PGF_2_ concentration of 34.0±2.7% and 48.1±3.4% respectively compared to the pre-EVH urinary 9α, 11β PGF_2_ pre-treatment concentration. No significant difference (p>0.05) was observed between pre-treatment and montelukast treatment for the pre-EVH urinary 9α, 11β PGF_2_ concentration.

The fish oil and combination treatment significantly reduced (p<0.05) the post-EVH urinary 9α, 11β PGF_2_ concentration by 17.1±2.2% and 17.8±2.5% compared to the post-EVH urinary 9α, 11β PGF_2_ pre-treatment concentration. However, no significant difference (p>0.05) was observed between pre-treatment and montelukast for the post-EVH urinary 9α, 11β PGF_2_ concentration.

### Nutrient Intake and Compliance

Subject adherence to the treatment regimens were assured by finding that pill counts at the end of each supplement period reflected that capsules were consumed regularly. Compliance as estimated from return-tablet count was high (median, 98%). Though usual diet was expected to vary between and among subjects, mean daily nutrient intake of the subject's diets did not differ significantly between the treatment regimens (p>0.05). (Table 3).

**Table 3:** Mean daily nutrient intake for each treatment period.

	Baseline (Pre-Treatment)*	Montelukast	Fish Oil	Combination Treatment
Alphatocopherol (mg)	10.4±1.5	9.7±2.2	8.9±1.9	9.1±1.3
Beta Carotene (mg)	4480±799	4900±1294	3196±523	4309±1063
Lycopene (mg)	9899±1726	7781±2167	8110±2710	7741±1147
Vitamin C (mg)	136.0±14.9	130.6±17.0	99.2±11.3	102±10.3
Magnesium (mg)	389.0±43.0	350±68.0	343±59.0	341±35.0
Sodium (mg)	3854±478	3239±605	3600±786	3211±367
Zinc (mg)	12.1±1.3	12.1±2.3	12.9±2.5	13.9±1.8
Monounsaturated Fatty Acids (g)	29.9±4.1	26.1±5.2	28.3±6.3	25.5±3.1
Omega-3 Fatty Acids (g)	1.5±0.2	1.4±0.2	1.3±0.3	1.3±0.2
Polyunsaturated Fatty Acids	16.5±2.1	14.8±2.7	15.1±3.2	13.8±1.8
Saturated Fatty Acids (g)	27.1±3.6	24.0±4.8	26.2±5.9	23.6±2.9
Protein (g)	96.1±12.6	83.2±14.9	91.9±21.4	80.8±8.1
Carbohydrates (g)	299.1±28.8	253.8±48.8	275.8±48.4	253.6±24.7
Fat (g)	79.7±10.3	70.7±13.6	75.6±16.3	68.2±8.2

Values are mean ± SEM. There were no significant differences (p>0.05) between groups for any specific nutrient.

*Food frequency measured from each subject on their usual diet.

## Discussion

This study has shown that fish oil and montelukast were both effective in reducing the airway reactivity to hyperpnea with dry air, however when fish oil was combined with montelukast no further attenuation in airway inflammation or hyperpnea-induced bronchoconstriction was observed. All three treatments significantly reduced bronchodilator use and improved post-exercise pulmonary function as demonstrated by the reduction in post-EVH FEV_1_ and FEF_25–75%_, and reduced the severity of hyperpnea-induced bronchoconstriction as measured by AUC_0–20_. Fish oil, montelukast and the combination treatment significantly reduced the fall in FEV_1_ post-EVH by approximately 49%, 37% and 41% respectively. We have previously shown that 3 weeks of fish oil supplementation (3.2 EPA and 2.0g DHA per day) reduced the fall in FEV_1_ at 15 minutes post-exercise by almost 80% in elite athletes with EIB [19], and reduced the post-exercise fall in FEV_1_ by approximately 64% in asthmatic subjects with EIB [18]. The degree of protection provided by montelukast on hyperpnea-induced bronchoconstriction in the present study is similar in magnitude to previous reports using an exercise challenge [11], [12], which have shown that 2-hr following a single 10-mg dose of montelukast the maximum fall in post-exercise FEV_1_ was reduced by approximately 52% [11] and 33% [12] compared to placebo treatment. In the present study symptom scores did not change as result of treatment. This is not unexpected since it is well documented that symptoms have poor predictive value for the diagnosis of EIB [34], [35].

It has been shown that airway pH is an important determinant of expired F_E_NO and airway inflammation, and thus there may be a casual relationship between airway acidification and airflow limitation in asthma [36]. EBC pH is lower in asthmatics and correlates positively with sputum eosinophilia, total nitrate/nitrite and oxidative stress, and shows good repeatability in asthmatics over a 1-year period without seasonal variation [37]. Airway acidity both accelerates human eosinophils necrosis and can cause the conversion of endogenous nitrate (NO_2_^−^) to nitric oxide (NO) and importantly, the acidic airway breath condensate pH in asthma normalizes with anti-inflammatory therapy [36], which supports our observation that all three anti-inflammatory treatments (fish oil, montelukast and combination treatment) significantly raised EBC pH compared to the more acidic pre-treatment value.

In the present study fish oil, montelukast and the combination treatment all reduced the pre-hyperpnea F_E_NO compared to the pre-treatment value in the EIB subjects, suggesting amelioration in baseline airway inflammation. Previous studies have shown that F_E_NO can be used as an indirect marker of asthmatic airway inflammation [38], and have shown a relationship between F_E_NO levels and EIB [39], [40], [41]. It has been shown that montelukast reduces F_E_NO in patients with mild and chronic asthma [38], [42], [43], suggesting an anti-inflammatory role for Cyst-LT_1_ receptor antagonism in asthma. Likewise, supplementation with omega-3 polyunsaturated fatty acids has been shown to reduce F_E_NO in patients with atopic asthma [44].

Eicosapentaenoic acid and DHA, derived from fish oil, can cause dual inhibition COX-2 and 5-LO pathways for metabolism of arachidonic acid. It has been shown that 5-LO product formation in the lung capillary bed is critically dependent on intravascular precursor fatty acid supply, with EPA representing the preferred substrate compared with arachidonic acid [45]. Eicosapentaenoic acid can inhibit release of arachidonic acid derived eicosanoids, thus reducing the generation of pro-inflammatory ‘tetraene’ 4-series LTs and 2-series prostanoids, and production of cytokines from inflammatory cells [46], and increasing the 5-series LTs [45]. Eosinophils, mast cells, basophils, and alveolar macrophages can directly synthesize the 4-series Cyst-LTs, which can increase vascular permeability and contract smooth muscle cells, causing bronchoconstriction, and may directly increase eosinophilic airway inflammation [18]. Although DHA may have similar anti-inflammatory effects as EPA, it does not act by direct competition with AA. DHA can decrease the release of AA from membrane phospholipids by decreasing phospholipase A2 activity [47], and decreasing the responsiveness of toll-like receptor-4 to LPS, thereby suppressing nuclear factor-kappaB activation and subsequent inflammatory gene transcription [48].

We recently examined the effects of pure EPA and pure DHA, and a variety of heterogeneous blends of EPA and DHA, on eicosanoid (i.e., leukotrienes and prostaglandins) and cytokine generation, and mRNA expression from LPS-stimulated human asthmatic alveolar macrophage cells [49]. The data indicate that the greater the EPA content of a fish oil formulation the greater the eicosanoid and cytokine reduction. In addition, a new class of mediator families derived from fish oil—the EPA- and DHA-derived resolvins (RvE1 and RvD1) and the DHA-derived protectin (PD1), which act locally, and possess potent anti-inflammatory novel bioactions have recently been indentified and may represent a potential mechanism for the therapeutic benefits derived from diets rich in these omega-3 essential fatty acids.

We have previously shown in elite athletes with EIB [19] that 3 weeks of fish oil supplementation (3.2 g EPA and 2.0 g DHA per day) increased tissue phospholipid n-3 PUFA concentration coincident with a significant suppression of urinary and blood eicosanoids (LTE_4_, 9α, 11β-PGF_2_, and LTB_4_, respectively) and pro-inflammatory cytokines (TNF-α and IL-1β). In a follow-up study [18] we found that sputum differential eosinophil, neutrophil, lymphocyte and macrophage cell counts and sputum supernatant concentrations of pro-inflammatory eicosanoids (LTC_4_-LTE_4_ and PGD_2_), and cytokines (TNF-α, IL-1β) were significantly reduced on a 3 week fish oil diet in asthmatic subjects with EIB. In addition, the amount of LTB_5_, a weak and partial antagonist compared with LTB_4_ in eliciting chemotactic and aggregating responses, generated from activated polymorphonuclear leukocytes was markedly increased following fish oil supplementation. The data from the present study and our previous observations [18], [19], [49] strongly suggest that dietary supplementation with fish oil can suppress airway inflammation in asthmatic subjects with EIB.

In the present study fish oil, montelukast and the combination treatment significantly reduced EBC Cyst-LT concentrations. Studies have shown a sustained increase in Cyst-LTs and other bronchoconstrictve eicosanoids, such as PGD_2_, in the airways after an exercise challenge [4], [50], and have been shown to be elevated for up to 6 hours after an exercise challenge in asthmatic subjects with EIB [4], [50]. Eosinophils, mast cells and basophils can directly synthesis Cyst-LTs, which can cause tissue edema, stimulate airway secretions, promote cell cycling and proliferation of airway smooth muscle, and may directly increase eosinophilic inflammation [24]. It has been shown that in some patient populations, the amount of eosinophilia in induced sputum is correlated with EIB severity [51]. Hallstrand et al. [52] have found that following an exercise challenge there was an increase in secreted phospholipase A_2_ group X (sPLA_2_ –X) protein in induced sputum and the percentage of epithelial cells immunostaining for sPLA2 –X in asthmatics with EIB. These findings [52] may provide an explanation for the elevated levels of pre-treatment EBC Cyst-LTs observed in the present study.

The inhibition of EIB by a Cyst-LT_1_ receptor antagonist alone is incomplete, which implicates other bronchoconstrictve mediators in the pathogenesis of EIB [53]. In the present study fish oil and the combination treatment, but not montelukast, significantly blunted the increase of 9α, 11β-PGF_2_ in urine following the dry gas hyperpnea challenge. In humans, PGD_2_ is a potent bronchoconstrictor [54] that is almost exclusively produced by mast cells [55]. 9α, 11β-PGF_2_, the urinary metabolite of PGD_2_, has been shown to be increased after allergen-induced bronchoconstriction [56], EIB [57], mannitol-induced bronchoconstriction [58] and hyperpnea-induced bronchoconstriction [25].

Since montelukast only blocks the bronchoconstrictor response to exercise by up to 70% [9], [10], [11], [12], [13], [14] it is perhaps surprising that both fish oil and montelukast had similar effects on the magnitude of attenuation of EIB and airway inflammation. A possible explanation for the apparently similar anti-inflammatory and bronchoconstrictor effects of fish oil and montelukast may be related to the dose and/or omega-3 fatty acid content of fish oil used in the current study. It is possible that a different concentration of EPA/DHA or a higher dose of fish oil may cause a greater suppression of EIB and airway inflammation; we have previously shown that the greater the EPA content of a fish oil formulation the greater the inhibition of the inflammatory response in human asthmatic alveolar macrophage cells [49]. However, the optimal daily intake and formulation of fish oil to be used to suppress EIB and airway hyperresponsiveness in asthma has not yet been determined.

Although both fish oil and montelukast alone significantly attenuated airway inflammation and hyperpnea-induced bronchoconstriction, treatment with fish oil and montelukast in combination did not provide a greater anti-inflammatory effect and suppression of bronchoconstriction than either treatment alone. A possible explanation for this observation may relate to the fact that fish oil feeding results in a decreased capacity of inflammatory cells to *synthesize* COX-2 and 5-LO-derived eicosanoids from arachidonic acid [59], which includes blocking the generation of Cyst-LTs from resident airway cells [18], [19], while montelukast, which is working further down the eicosanoid metabolic pathway, only blocks the receptor that binds the Cyst-LTs.

The present study has several limitations. First, the absence of a non-active treatment arm (double-dummy) in the study design, and the absence of statistical power for equivalence testing between fish oil supplementation and montelukast, is a weakness that should be acknowledged. Second, the included patients were asthmatic with documented HIB and therefore the conclusions drawn from this small proof-of-concept study should not be generalized to all individuals with asthma.

In conclusion, while we have demonstrated that fish oil and montelukast are both effective in attenuating airway inflammation and hyperpnea-induced bronchoconstriction in asthmatic subjects, treatment with fish oil and montelukast in combination does not provide a greater anti-inflammatory effect or suppression of bronchoconstriction than either treatment alone. It is clear that fish oil supplementation should be considered as an alternative treatment for EIB. Importantly, fish oil, rich in EPA and DHA, holds promise for providing relief from pathologic airway responses by serving as pro-resolving agonists and/or inflammatory antagonists in stimulating a return to respiratory homeostasis in asthma and EIB.

## Supporting Information

Figure S1 Flow of participants through the study.
(0.12 MB TIF)

Protocol S1 (0.02 MB DOCX)

Checklist S1 CONSORT 2010 Checklist of Information to Include When Reporting a Randomized Trial.
(0.22 MB DOC)
