# Supplementary figures and images for: Randomized Controlled Trial of Fish Oil and Montelukast and Their Combination on Airway Inflammation and Hyperpnea-Induced Bronchoconstriction
Source: PLoS One. 2010 Oct 18;5(10):e13487. doi: 10.1371/journal.pone.0013487 (PMC2956690; doi:10.1371/journal.pone.0013487)

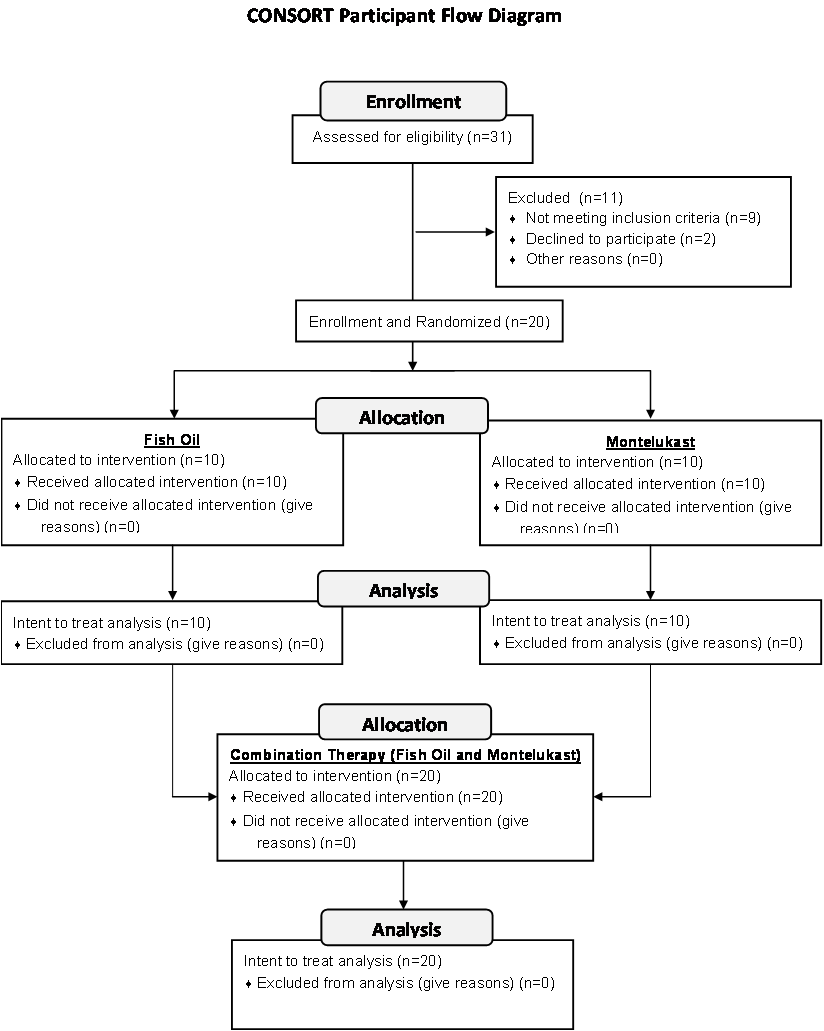

Supplement: Figure S1 — Flow of participants through the study. (0.12 MB TIF) [file pone.0013487.s001.tif]
