# Supplementary material for: Randomized Controlled Trial of Fish Oil and Montelukast and Their Combination on Airway Inflammation and Hyperpnea-Induced Bronchoconstriction
Source: PLoS One. 2010 Oct 18;5(10):e13487. doi: 10.1371/journal.pone.0013487 (PMC2956690; doi:10.1371/journal.pone.0013487)
Supplement: Protocol S1 — (0.02 MB DOCX) [file pone.0013487.s002.docx]

**CONSORT Participant Flow Diagram**

**Combination Therapy (Fish Oil and Montelukast)**

Allocated to intervention (n=20)

♦ Received allocated intervention (n=20)

♦ Did not receive allocated intervention (give reasons) (n=0)

## Enrollment

Excluded (n=11)

♦  Not meeting inclusion criteria (n=9)

♦  Declined to participate (n=2)

♦  Other reasons (n=0)

Assessed for eligibility (n=31)

Enrollment and Randomized (n=20)

Intent to treat analysis (n=20)
♦ Excluded from analysis (give reasons) (n=0)

## Analysis

## Allocation

Intent to treat analysis (n=10)
♦ Excluded from analysis (give reasons) (n=0)

Intent to treat analysis (n=10)
♦ Excluded from analysis (give reasons) (n=0)

## Analysis

**Montelukast**

Allocated to intervention (n=10)

♦ Received allocated intervention (n=10)

♦ Did not receive allocated intervention (give reasons) (n=0)

**Fish Oil**

Allocated to intervention (n=10)

♦ Received allocated intervention (n=10)

♦ Did not receive allocated intervention (give reasons) (n=0)

## Allocation
